# Surgical Strategy for Anterior Tibial Plateau Fractures in Hyperextension Knee Injuries

**DOI:** 10.1111/os.12997

**Published:** 2021-04-04

**Authors:** Zhong‐yu Liu, Jin‐li Zhang, Chang Liu, Qing Cao, Qi‐jie Shen, Jun‐chao Zhao

**Affiliations:** ^1^ Department of Orthopaedics Tianjin Hospital Tianjin China

**Keywords:** Anterior tibial plateau, Approaches, Fracture, Internal fixation, Surgical treatment

## Abstract

**Objective:**

The aim of the present study was to summarize the clinical characteristics, treatment strategies, and clinical results for anterior tibial plateau fractures caused by hyperextension injuries.

**Methods:**

We performed a retrospective analysis of 26 cases of anterior tibial plateau fractures that were treated with open reduction and internal fixation from January 2016 to December 2019, including 16 men and 10 women, aged 26–68 years old, with an average age of 47 ± 12.5 years. According to the three‐column theory classification, there were 16 cases of single‐column fractures (9 cases of anteromedial fractures and 7 cases of anterolateral fractures), 3 cases of two‐column fractures (anteromedial + anterolateral fractures), and 7 cases of three‐column fractures. Options for the surgical approach included anteromedial, anterolateral, modified anterior median, and anterolateral + posteromedial incision. The implants included a T‐shaped plate, an L‐shaped plate, a horizontal plate, and a TomoFix plate. The surgical approach and fixation method were selected based on the characteristics of the anterior tibial fracture. The Rasmussen radiological criteria were used to evaluate the effects of fracture reduction and fixation. The knee joint function was evaluated using the knee function evaluation criteria of the Hospital for Special Surgery. Medial and lateral stress tests, the Lachman test, and the pivot shift test were used to evaluate the stability of the knee joint. The range of knee motion was recorded.

**Results:**

All cases were followed up for 12–24 months, with an average follow up of 15.7 months. The operation time was (148 ± 42) min; the intraoperative blood loss was (150 ± 50) mL. A total of 22 cases were anatomically reduced and 4 cases were well‐reduced, and the compression reduction rate was 100%. According to the Rasmussen radiology scoring, 17 cases were excellent and 9 cases were good. The excellent and good rate was 100%. The fracture healing time was 3.3 months. There is no difference in fracture healing time for different fracture types. Both the Lachman and pivot shift test findings were normal in 24 patients and nearly normal in 2 patients. The posterior drawer test was normal in 25 patients and close to normal in 1 patient. The varus stress test was normal in 24 patients and nearly normal in 2 patients, while the valgus stress test was normal in 23 patients, nearly normal in 2 patients, and abnormal in 1 patient. The range of motion (ROM) was 100°–137°, with an average of 125° ± 11.7°. The Hospital for Special Surgery (HSS) knee score at the last follow up was 79–98 points, with an average of 87.54 ± 8.36 points; the results were excellent in 21 cases and good in 5 cases. Therefore, 100% of results were excellent or good. Two cases had superficial wound infections after the operation. The recovery of 2 patients with common peroneal nerve injury was poor.

**Conclusion:**

The appropriate surgical approach and fixation method were performed according to the different positions of the anterior tibial fracture and satisfactory results were obtained after surgery.

## Graphical abstract

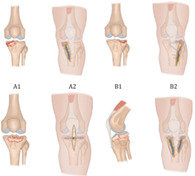

(A1) Anteromedial tibial plateau fracture. (A2) The anterior medial approach and T‐shaped locking plate fixation were used. (B1) Anterolateral tibial plateau fracture. (B2) Anterolateral incision was used and the simple anterior compression of the tibia was fixed with the horizontal plate. If combined with a split fracture, an L‐shaped plate was used. (C1) Anteromedial + anterolateral fractures. (C2) The modified anterior median approach was used. The fixation was used with the horizontal plate. (D1) Tibial plateau hyperextension three‐column fractures. (D2) Both anterolateral and posteromedial approaches were used. The fixation included a T‐shaped plate and an L‐shaped plate.

## Introduction

Tibial plateau fractures are common clinically. The most common injury mechanism is the knee joint being injured by force in the extension or flexion position. However, some tibial plateau fractures are caused by axial force in the knee joint hyperextension position, resulting in depression of the anterior tibial plateau and soft tissue injury^1^. This type of injury is more serious, and the injury mechanism and treatment differ to those of extension and flexion tibial plateau fractures^2^, ^3^. These fractures are now referred to as “hyperextension tibial plateau fractures” because hyperextension plateau fractures often lead to fractures of the anterior rim of the tibia combined with significant ligament injuries, leading to instability of the knee joint. The incidence of injury to soft tissues, such as nerves and blood vessels, is high, which can seriously endanger the survival of the limbs^4^, ^5^. Because of the poor functional recovery, its diagnosis and treatment have been receiving increasing attention.

Hyperextension tibial plateau fractures are rare, but the injury is serious. Because the knee joint is subjected to hyperextension violence, the femoral condyle compresses the anterior tibial plateau, resulting in depression of the anterior tibial plateau, sometimes combined with loss of posterior slope of the tibial plateau^6^. Many scholars have also reported on anterior tibial plateau fractures combined with injuries to the posterior cruciate ligament (PCL) and posterolateral structures^7^, ^8^. Such fractures often require surgical treatment due to damage to important ligaments around the knee joint at the same time, causing knee joint instability^9^. Due to the lack of understanding of this type of fracture in the past, it was easy to overlook the need for reduction and fixation of anterior plateau tibial fractures, leading to knee instability later on.

For hyperextension tibial plateau fractures, the treatment involves the reduction of fractures while repairing important ligament injuries, restoring the stability of the knee joint. If the depression involves the tibial rim, the tibial rim should be reconstructed, restoring the stability of the joint^10^. The anterior edge of the tibia is characterized by a small amount of less soft tissue coverage on the front of the tibia, and the patellar tendon runs directly in front of the tibia. At present, the fracture characteristics of anterior tibial fractures caused by knee hyperextension injuries are unclear. There is no consensus on the ideal choice of approach and fixation methods for anterior tibial plateau fractures^11^. For anteromedial tibial plateau fractures, the T‐shaped plate is usually used for fixation, and satisfactory results are obtained. However, if the fracture is anterolateral to or behind the patellar tendon, no plate is available. Some authors have used Kirschner wire fixation or screw fixation, but the fixation strength is poor and does not provide sufficient stability for the fracture. Determining how to reduce and fix the fracture of the anterior tibial plateau caused by the hyperextension injury is still a clinical problem that needs to be resolved.

Therefore, the present study retrospectively analyzes the data of patients with hyperextension injuries of the knee joint with fractures of the anterior tibia plateau. Preoperative and postoperative imaging data are examined, and functional outcomes are evaluated. The purpose of the present study is to examine: (i) the characteristics of anterior tibial plateau fractures; (ii) the choice of surgical approaches for hyperextension anterior tibial plateau fractures; and (iii) the fixation methods for anterior tibial fractures resulting from hyperextension injuries.

## Materials and Methods

### Inclusion and Exclusion Criteria

The inclusion criteria were: (i) a closed hyperextension injury of the knee joint combined with a fracture of the anterior tibia; (ii) treatment with open reduction and internal fixation; (iii) no popliteal artery damage; (iv) Rasmussen radiological criteria were used to evaluate the effect of fracture reduction and fixation, and the knee function score of the HSS score was used to evaluate the surgical effect; and (v) this was a retrospective case analysis.

The exclusion criteria were: (i) no hyperextension; (ii) open fractures; (iii) adolescents with unclosed epiphyses; (iv) previous knee disease or limited joint function; (v) pathological fractures; (vi) multiple fractures of the lower limbs, affecting the subsequent rehabilitation of the knee joint; and (vii) follow‐up time <12 months.

### General Information

From January 2016 to December 2019, a total of 26 patients with anterior tibial plateau fractures were included in the present study based on the above inclusion and exclusion criteria. The study included 16 male and 10 female patients; patients were aged 26–68 years, with an average age of 47 ± 12.5 years. In terms of the causes of injury, there were 15 cases of traffic accidents, 8 cases of crush injuries, and 3 cases of high‐level fall injuries. According to eh Luo *et al*.^12^ three‐column classification, there were 19 cases of single‐column fractures, including 9 cases of anteromedial fractures and 7 cases of anterolateral fractures, 3 cases of two‐column fractures (anteromedial + anterolateral fractures, and 7 cases of three‐column fractures. According to Schatzker classification^13^, there were 6 type II cases, 9 type IV cases, 5 type V cases, and 6 type VI cases.

In this group, there were 2 cases of patellar fractures and 3 cases of fibular capitulum fractures. Among the ligament injuries of the knee joint, there were 2 cases of anterior cruciate ligament (ACL) injuries, 4 cases of PCL injuries, 3 cases of ruptures of the medial collateral ligament (MCL), 4 cases of lateral collateral ligament (LCL) injury, 7 cases of posterolateral complex (PLC) injury, 2 cases of PLC + PCL injury, and 7 cases of lateral meniscus injury.

In 2 patients with common peroneal nerve injuries, ankle joint dorsiflexion was absent and dorsal sensation had decreased. Another 2 patients with compartment syndrome experienced ipsilateral calf swelling, toe traction pain, and a mobility disorder.

### Preoperative Preparation and Operative Strategy

**Fig 1:**
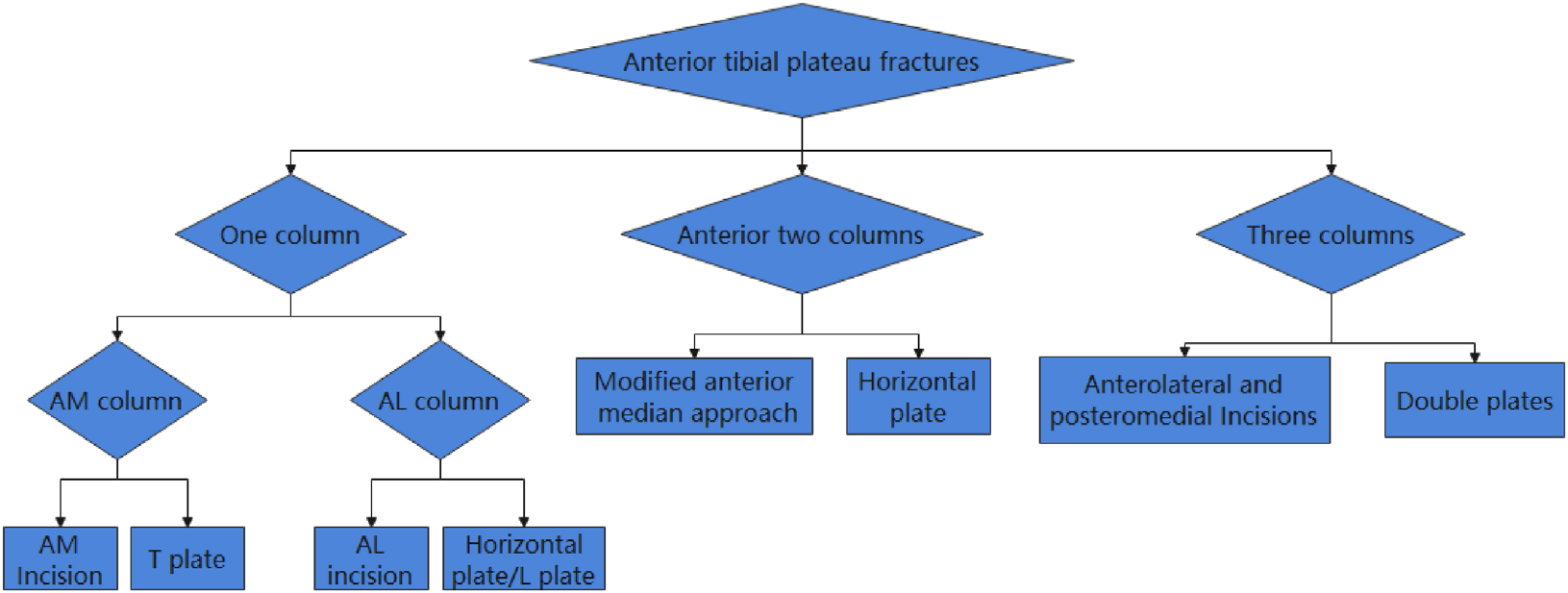
Surgical strategy for anterior tibial fractures. AM, anteromedial; Al, Anterolateral.

All patients underwent X‐ray, 3D CT, and MR examination before surgery to understand the type of fracture, the location of the fracture and the comminuted displacement, the degree of compression of the joint surface, and the soft tissue injury. Preoperatively suspected popliteal arteriovenous injuries require vascular Doppler ultrasound or CT angiography. The characteristics of tibial plateau fractures were analyzed in detail. We determined the surgical approach and method of internal fixation to use based on the fracture characteristics and the extent of involvement. For simple anteromedial fractures, the anteromedial approach to the knee joint can be used for fixation with a T‐shaped plate. For simple anterolateral fractures, the parapatellar tendon lateral approach can be used for fixation with a horizontally‐oriented rim plate. For anteromedial + anterolateral fractures, using a modified anterior median approach, incisions were made on both sides of the patellar tendon and fixed with a horizontally‐oriented rim plate. For three‐column fractures, the fractures were fixed with an L‐shaped plate and a TomoFix plate (see Fig. 1).

All cases were treated with detumescence, pain relief, and low molecular weight heparin to prevent deep venous thrombosis of the lower limbs. Time from injury to surgery was 7–29 days, with an average of 11.8 ± 5.9 days.

### Surgical Method

General anesthesia or continuous epidural anesthesia was used. The patient was placed in the supine position. A pneumatic tourniquet was used to control intraoperative bleeding. The tourniquet pressure was maintained at 370 kpa.

#### Anteromedial Tibial Plateau Fractures

**Fig 2:**
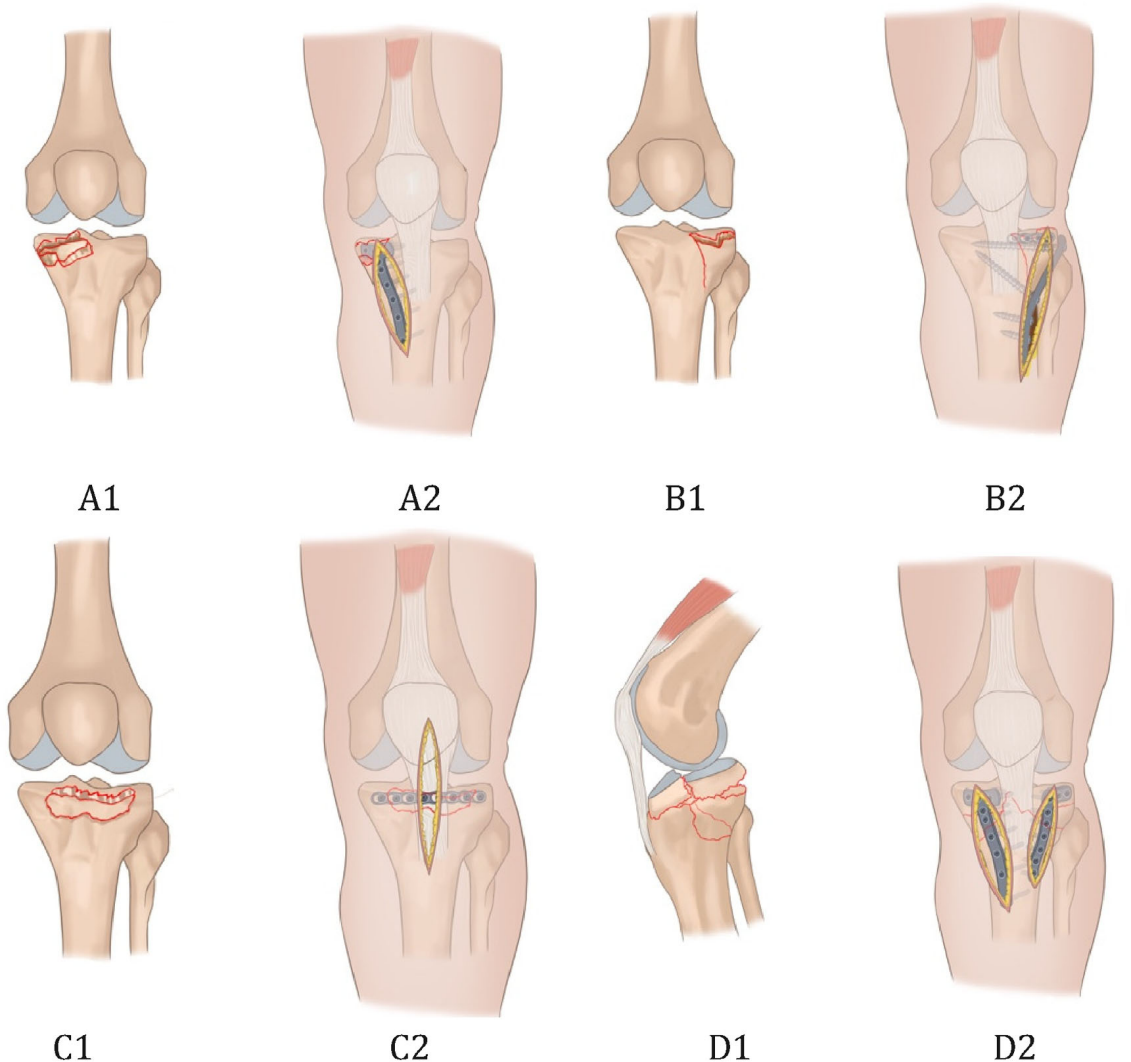
A1, Anteromedial tibial plateau fracture. A2, anterior medial approach and T‐shaped locking plate fixation were used. B1, anterolateral tibial plateau fracture. B2, Anterolateral incision was used, the simple anterior compression of tibia is fixed with the horizontal plate. If combined with the split fracture, an L‐shaped plate was used. C1, Anteromedial + anterolateral fractures. C2, A modified anterior median approach was used. The fixation was used with the horizontal plate. D1, Tibial plateau hyperextension three‐column fractures. D2, Anterolateral and posteromedial approaches were used. The fixation included a T‐shaped plate and an L‐shaped plate.

1. Incision: An approximately 8‐cm‐long longitudinal incision was made on the anterior medial side of the knee joint. The anteromedial incision originated from the medial patellar tendon of the lower part of the patella and was extended along the tibia to the distal end.
2. Exposure: The subcutaneous and deep fascia were cut sequentially to protect the medial collateral ligament, and the pes anserinus tendon was peeled off under the periosteum. The medial anterior joint capsule was cut transversely.
3. Reduction: The fracture and meniscus injury were checked. The compressed articular surface fragments were restored under direct vision, the normal posterior slope was restored, and the Kirschner wire was temporarily fixed. The anteromedial wall fracture was also reduced, and the anteromedial cortical support was restored. Bone defects under the articular surface were filled with artificial bone graft. The fracture reduction was confirmed with the C arm X‐ray machine.
4. Fixation: The T‐shaped locking plate was used to fix the medial fragment, and the distal end of the plate was passed through the deep surface of the pes anserinus tendon (see Fig. 2 A1,2).

#### Anterolateral Tibial Plateau Fractures

1. Incision: The anterolateral incision originated from the posterolateral aspect of the lower part of the patella and was extended along the tibia to the distal end via the Gerdy nodule.
2. Exposure: The anterolateral incision was cut layer by layer, the anterolateral tibial plateau was separated and exposed, the joint capsule was opened, the meniscus was examined, the meniscus was retracted and protected, and the anterolateral fracture block and articular surface were exposed.
3. Reduction: The fracture and meniscus injury were checked. The compressed articular surface fragments were reduced under direct vision. Temporary fixation was performed with the Kirschner wires. If combined with an anterolateral wall split fracture, the split fracture was opened, the compressed fracture was reduced, and the lateral wall fracture was also reduced. Bone defects under the articular surface were filled with artificial bone graft.
4. Fixation: A 2.7‐mm anatomical locking plate system (Weiga, China) was bent to follow the lateral joint outline, and the articular surface fragments were fixed by multi‐angle screws, so that the comminuted lateral articular surface fragments were fixed as a whole. If combined with an anterolateral split fracture, the anterolateral fracture was fixed with the L‐shaped tibial plateau locking plate and screws (see Fig. 2 B1,2).

#### Anteromedial + Anterolateral Tibial Plateau Fracture

1. Incision: A modified anterior median approach was used. The incision originates from the patella and was extended along the patellar tendon to the distal end via the tibial tubercle laterally.
2. Exposure: The skin and superficial fascia were cut, and the two windows were made longitudinally on the medial and lateral sides of the patellar tendon. The patellar tendon was stretched and observed through two windows.
3. Reduction: The anterolateral and posterolateral compression of the articular surface fragments were restored through two windows. The Kirschner wire was used for temporary fixation, and the fracture reduction was determined under direct vision or by C‐arm fluoroscopy.
4. Fixation: Bent plates were attached to the lateral joint edge of the tibial plateau, and the articular surface fragments were fixed by multi‐angle screws. (see Fig. 2 C1,2)

#### Hyperextension Three‐Column Fracture

1. Incision: Two surgical approaches were made, anterolateral and posteromedial to the proximal tibia. The posteromedial approach allows visualization of the posterior tension fracture for reduction and placement of an anteromedial plate.
2. Exposure: The medial approach is required to expose the posteromedial fracture of the tibia.
3. Reduction: Reduction of the articular surface of the tibial plateau articular surface was performed as well as restoration of sagittal plane alignment through elevation of the anterior metaphysis.
4. Fixation: Our preferred method of fixation involved an L‐shaped plate in the lateral tibia and a Tomofix plate in the anteromedial tibia (see Fig. 2 D1,2).

### Treatment of Other Damage

The patella fracture was open reduced and fixed with a tension band. After the completion of open reduction and internal fixation, the varus and valgus stress tests were used to assess the stability of the involved knee joint, under the guidance of C‐ arm fluoroscopy. If the knee joint space on the involved side was more than 5 mm wider than on the contralateral side, the possibility of collateral ligament injury was considered. Then, the collateral ligament injury was repaired with a suture anchor. If the ACL and the PCL were completely ruptured, arthroscopic reconstruction was performed using the single‐bundle technique. For PCL reconstruction, an artificial ligament (Lars, Dijon, France) was used as the graft. The autogenous tendons were used in the ACL reconstruction. For meniscus tears along the edge of the joint capsule, suturing was performed. If there is meniscal bucket handle or horizontal tear, a partial meniscectomy should be performed to retain the residual meniscus.

### Postoperative Treatment

The dressing was pressure‐wrapped after the wound was sutured. Pain relief and anti‐inflammatories were given postoperatively for symptomatic treatment. Low molecular weight heparin was given to patients to prevent deep vein thrombosis at 12 h after surgery and for 5 weeks thereafter. Postoperatively, the leg was immobilized with a hinged knee brace in full extension. Isometric quadricep‐strengthening exercises, straight‐leg raises, and patellar mobilization exercises were started during the second postoperative day. Passive knee flexion was started at 2 weeks postoperatively, while active knee flexion was allowed at 4 weeks postoperatively. Partial weight‐bearing was allowed at 6 weeks postoperatively and full weight‐bearing was allowed at 8 weeks postoperatively. Active knee flexion was avoided for the first 6 weeks. Two patients underwent ACL reconstruction 6 months after the first operation due to ACL rupture. All other patients were allowed to return to normal activities 6 to 9 months postoperatively.

### Follow‐up and Efficacy Evaluation Indicators

The outpatients were reviewed 2, 6, and 12 weeks after surgery, and were followed up monthly. The fixation and healing of the fracture were observed by X‐ray and 3D CT examination. Summarize the patient's fracture healing time, fracture reduction, knee stability, knee function score, knee range of motion, and postoperative complications.

#### Rasmussen Radiological Evaluation

The Rasmussen radiological criteria were used to assess the fracture reduction and fixation at the last follow up^14^. The assessment includes consideration of whether the joint surface is compressed, whether the tibial plateau has widened, and whether there is a knee varus or valgus deformity; a score of 18 is excellent, 12–17 is good, 6–11 is acceptable, and <6 is poor.

#### Evaluation of Hospital for Special Surgery Function

The Hospital for Special Surgery (HSS) knee function evaluation criteria are used for knee joint function assessment^15^. The HSS score reflects pain, functional mobility, muscle strength, knee deformity, stability, and other items, indicating the overall function of the knee joint surgery; a score of 85–100 is excellent, 70–84 is good, 60–69 is acceptable, and below 60 is poor.

#### Assessment of Knee Stability

Knee stability was assessed by physical examination, using the Lachman test, the pivot shift test, the anterior and posterior drawer test, and the varus and valgus stress test. The anterior, posterior, and rotational stability were evaluated using the Lachman and pivot shift tests. The range of knee motion was recorded. For the Lachman test, patients were placed in the supine or prone position, with knee flexion of 30°. Comparison was made with the healthy side and positive results suggested ACL or PCL injury. For the pivot shift test, the patient was placed in a supine position, the knee joint was straightened, the ankle joint was rotated, and the knee joint was everted. When the knee was flexed 30°– 40°and the tibia was suddenly reset, the result of the test was positive was positive. ACL and PCL were evaluated through the anerior and posterior drawer test. The patients were placed in supine position with knee flexion of 80° to 90° and their feet fixed on the table. The proximal tibia moved forward or backward relative to the femur. Varus and valgus stress tests were used to evaluate the stability of the lateral and medial structures.

### Statistical Analysis

Statistical analysis was performed using SPSS version 12.0 (SPSS, Chicago, IL, USA). Postoperative outcomes were retrieved and analyzed. Data are presented as mean ± standard deviation. Comparison of the four groups of measurement data was performed using a multiple independent samples *t*‐test, and the difference was statistically significant at *P* < 0.05.

## Results

### The Operation

#### General Situation

All 26 patients in this group successfully completed the operation. The operation time was 148 ± 42 min (range: 76 – 195 min); the intraoperative blood loss was 150 ± 50 mL (range: 30 – 300 mL).

#### Internal Fixation of Tibial Fractures

**Fig 3:**
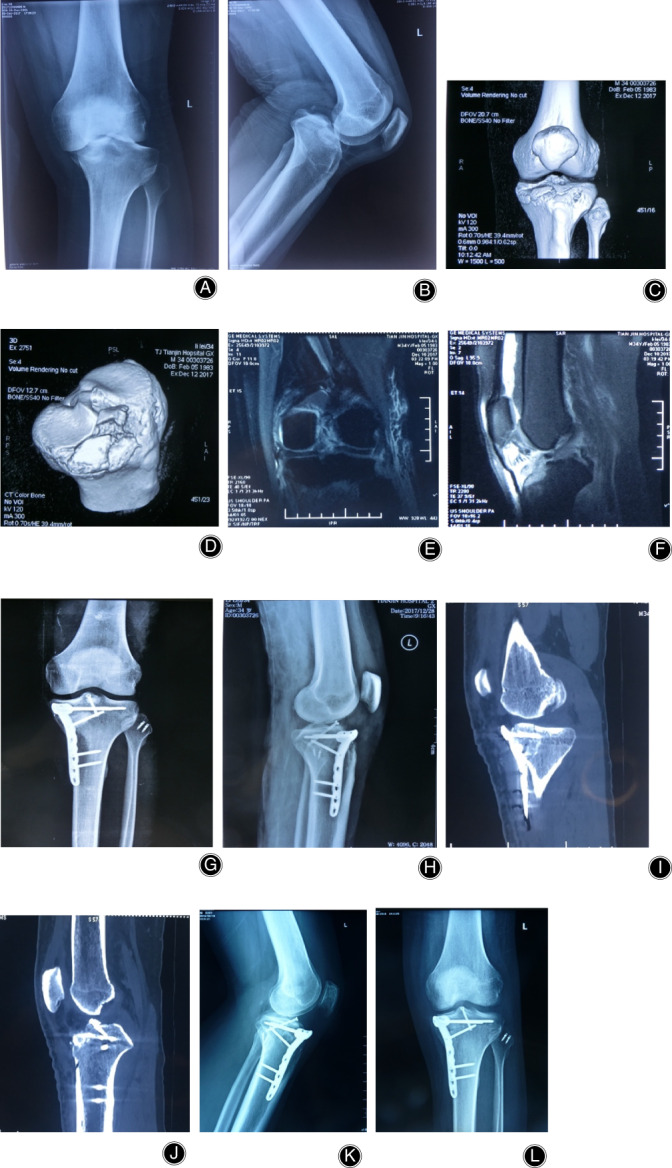
Anteromedial tibial plateau fracture. Male, 34 years old. (A, B) X‐Ray shows that the medial tibial plateau had a displaced fragment and location of the knee. (C, D) 3D CT showed the anteromedial tibial plateau fracture, compression of the articular surface, and avulsion fracture of tibial intercondylar eminence. (E, F) MRI showed the lateral collateral ligament (LCL) injury and avulsion fracture of the ACL. (G, H) The anteromedial articular surface were reduced and fixed with “T” locking plates. The fracture of the tibial intercondylar eminence was fixed with screws. The LCL injury was repaired with anchoring sutures. (I, J), Postoperative CT showed anatomical reduction of the fracture. (K, L) The fracture healed with anteromedial reduction 1 year after the operation.

**Fig 4:**
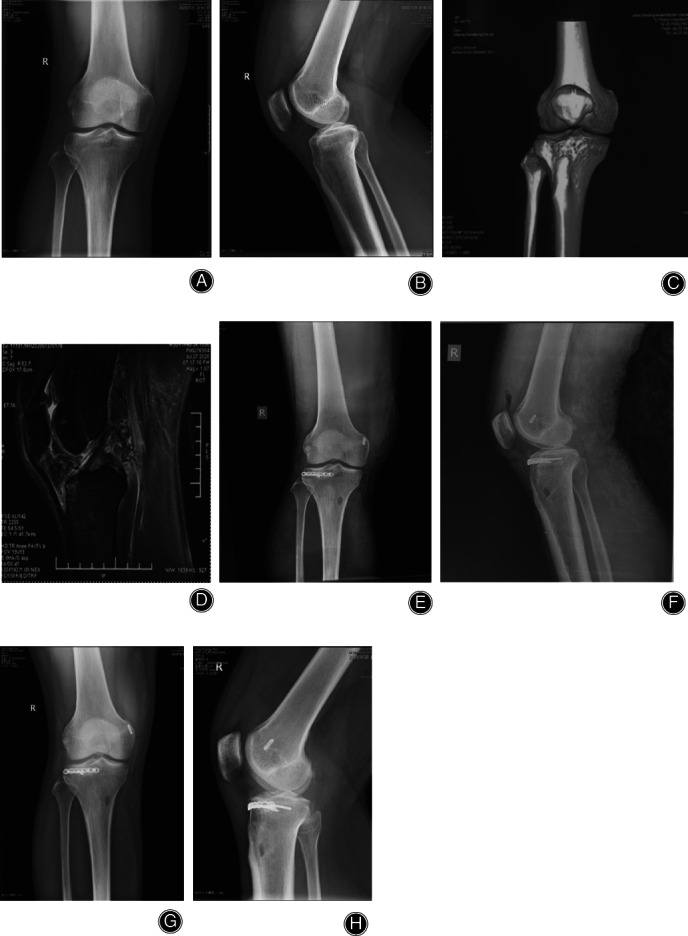
Hyperextension bicondylar fracture. Male, 42 years old. (A, B) No fracture can be seen on X‐Ray. (C) 3D CT showed the compression of the anterolateral tibial plateau. (D) MRI showed the posterior cruciate ligament (PCL) injury. (E, F) The anterolateral articular surface was reduced and fixed with a horizontally‐oriented rim plate; arthroscopic reconstruction of the PCL was performed using the single‐bundle technique. (G, H) The fracture healed and satisfactory reduction had been achieved 1 year postoperatively.

**Fig 5:**
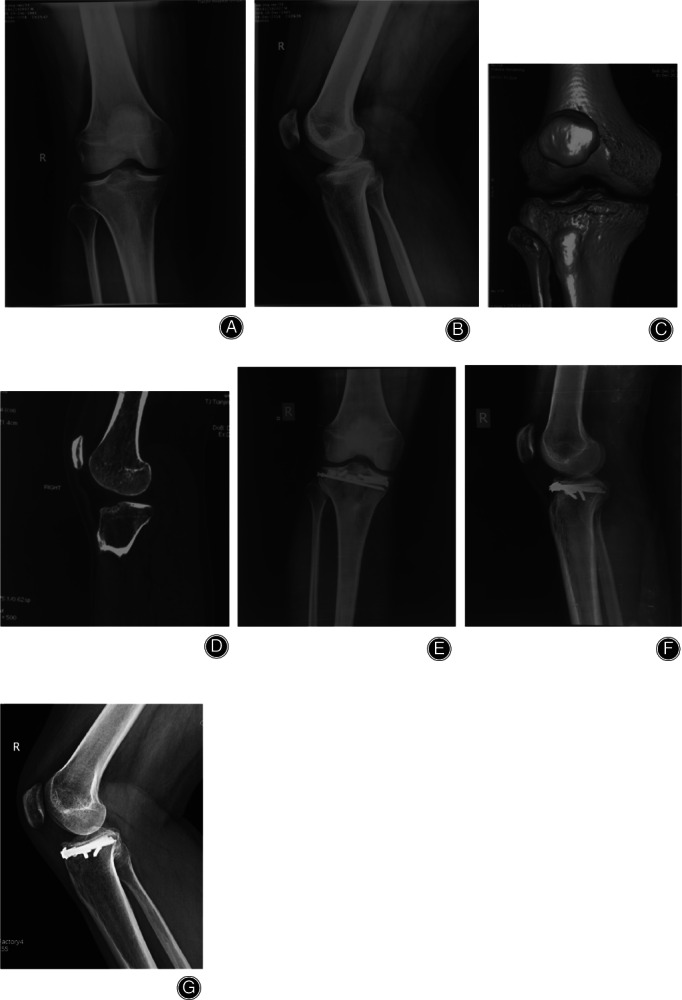
Anteromedial + anterolateral tibial plateau fracture. Male, 33 years old. (A, B) X‐ray shows that no fracture could be seen. (C, D) 3D CT showed the compression of anteromedial + anterolateral tibial plateau, which was located behind the patellar tendon. (E, F) The fracture were reduced and fixed with a horizontally‐oriented rim plate. (G), The fracture healed and satisfactory reduction had been achieved 1 year postoperatively.

**Fig 6:**
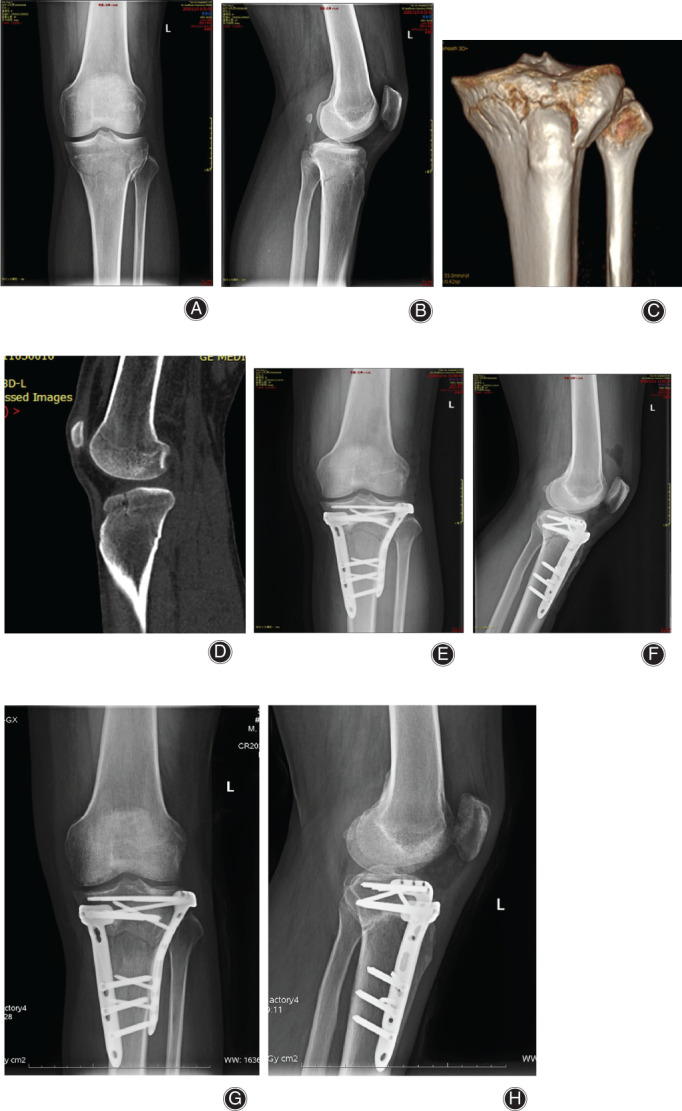
Anteromedial + anterolateral tibial plateau fracture. Male, 33 years old. (A, B) X‐ray shows medial and lateral tibial plateau fracture, and the loss of posterior slope of tibial plateau. (C, D) 3D CT shows the compression of anteromedial and anterolateral tibial plateau, and posterior tension fracture. (E, F) The fracture was reduced and fixed with an L‐shaped plate in the lateral tibia and a Tomofix plate in the anteromedial tibia. (G, H) The fracture healed and satisfactory reduction was achieved 6 months postoperatively.

All 26 cases were treated with open reduction and internal fixation. Nine patients in the simple anteromedial tibial plateau fracture group were treated with an anteromedial T‐shaped plate (Synthes, Switzerland; a typical case is shown in Fig. 3). Of the 7 cases in the simple anterolateral tibial plateau fracture group, 4 with lateral splitting fractures were fixed with an L‐shaped lateral plate (Synthes, Switzerland). Three cases of simple anterolateral compression of the tibia and 3 cases that were posterior to the patellar tendon (anterior medial + anterolateral) were fixed with a horizontal plate (Weigao,China; typical cases are shown in Figs 4 and 5). Seven cases of tibial plateau hyperextension three‐column fractures were treated with an L‐shaped plate (Synthes, Switzerland) in the lateral tibia and a Tomofix plate (Synthes, Switzerland) in the anteromedial tibia. (a typical case is shown in Fig. 6).

#### Treatment of Other Injuries of the Knee Joint

Of the 26 cases in this group, 2 cases had a patella fracture, and the patella was reduced and fixed with a steel wire tension band. Three cases had avulsion fractures of the fibula. Two of three cases of fibular capitulum fractures were fixed with a screw. Another case was treated conservatively without displacement. If the medial and lateral collateral ligaments were found to be damaged by preoperative MR examination, the stability of the knee joint during the operation was checked after fracture reduction. Among them, 3 cases with medial collateral ligament and femoral peel off injuries were repaired with anchoring sutures during surgery; 5 cases with PLC injuries were repaired with anchoring sutures. In 7 patients with partial injury of the PCL, the PCL was reconstructed in 2 patients during the operation. In 2 patients with an ACL injury, reconstruction was performed in the second stage of the operation. Among the 7 cases of meniscus injury, partial meniscectomy was performed in 3; 4 cases of longitudinal tears of the meniscus were repaired by suturing.

#### Bone Grafting

In this group of 11 patients, the bone defect below the articular surface after the reduction of the lateral tibial plateau compression area was filled with injectable calcium sulfate MIIGTMX3 (Wright, USA) bone material.

### Fracture Healing

All cases in this group were followed up for 15.7 months (range: 12–24 months). All fractures achieved bone union, and the fracture healing time was 3.3 months (range: 3–6 months). There was no difference in fracture healing time for different fracture types.

### Radiological Evaluation

In the evaluation of postoperative fracture reduction, it was determined that 22 cases of the joint surface were anatomically reduced and 4 cases were well‐reduced (articular surface compression 2 – 5 mm). The reduction of tibial plateau fractures was evaluated using the Rasmussen radiology score. Among them, 17 cases were excellent and 9 cases were good. The excellent and good rate was 100%.

### Knee Joint Function Evaluation

#### Knee stability

Both the Lachman and pivot shift test findings were normal in 24 patients and nearly normal in 2 patients. The posterior drawer test was normal in 25 patients and nearly normal in 1 patient. The varus stress test was normal in 24 patients and nearly normal in 2 patients, while the valgus stress test at 30° of flexion was normal in 23 patients, nearly normal in 2 patients, and abnormal in 1 patient.

#### Functional Outcomes

The patient's knee joint extended 0°and flexed 100 ± 8.2°at 6 weeks postoperatively. Twelve months after surgery, the range of motion (ROM) was 100°–137°, with an average of 125° ± 11.7°. The HSS knee score at the last follow up was 79–98 points, with an average of 87.54 ± 8.36 points; the results were excellent in 21 cases and good in 5 cases. Therefore, 100% of results were excellent or good. At the final follow up, all patients were able to return to normal daily life and 21 patients were working.

#### Comparison of different fracture groups

The 26 cases in this group were divided into four groups: a simple anteromedial column fracture group, a simple anterolateral column fracture group, an anteromedial + anterolateral fracture group, and a hyperextension three‐column fracture group. In the four groups, there was no significant difference in the postoperative fracture healing time, the Rasmussen score, the knee movement, and the HSS score. (Table 1).

**TABLE 1:** Comparison of postoperative outcomes in four subgroups (*Mean±SD*)

Groups	Number	Healing time (week)	Rasmussen score	Knee movement (°)	Knee stability	HSS score
AM column	9	12.5	16.21 ± 1.77	126 ± 9.1	Stability	86.34 ± 6.59
AL column	7	13.23	16.24 ± 1.82	129 ± 11.9	Stability	87.86 ± 9.40
AM+AL column	3	12.54	16.21 ± 1.91	127 ± 10.7	Stability	89.33 ± 5.37
Three columns	7	13.56	16.01 ± 1.27	121 ± 11.3	Stability	84.47 ± 4.51
*P*‐value	‐	0.433	0.317	0.614	—	0.637

AL, anterolateral; AM, anteromedial; HSS, Hospital for Special Surgery.

#### Complications

Two cases had superficial wound infections after the operation. The wounds healed after 2 weeks. One case had limited knee flexion, with 0° extension and only 100° flexion. The patient did not require further treatment. One patient with traumatic arthritis was given symptomatic treatment with non‐steroidal anti‐inflammatory drugs, and pain symptoms were relieved after reduced weight‐bearing activities.

The recovery of 2 patients with common peroneal nerve injuries was poor. Their sensory function partially recovered, their ankle dorsiflexion function did not recover significantly, they could walk with the assistance of a brace, and their gait was restricted. There was no implant breakage or loosening in this group, no postoperative compartment syndrome occurred, and no postoperative complications such as vascular and nerve injury occurred.

## Discussion

### Characteristics of Anterior Tibial Plateau Fractures

Hyperextension tibial plateau fractures are caused by hyperextension violence of the knee joint, and they commonly have a similar compression fracture pattern to anterior tibial plateau fractures. However, in the case of hyperextension injuries directly on the tibia, the knee joint can be accompanied by varus or valgus, internal or external rotation, or a neutral hyperextension injury^16^. Based on the direction of force, hyperextension tibial plateau fractures are usually divided into simple hyperextension fractures, hyperextension valgus fractures, and hyperextension varus fractures^2^. Varus violence can cause anteromedial tibial fractures. Valgus violence causes anterolateral tibia fractures, as well as simultaneous anterior internal and external fractures. According to the position of the fracture, fractures can be divided into simple anterior tibial compression fractures and hyperextension bicondylar fractures. Hyperextension bicondylar fractures are due to excessive force. The fracture line continues to the fracture of the posterior cortical bone, which is characterized by compression of the anterior tibial plateau combined with a tension fracture of the posterior tibia and loss of the posterior slope of the tibial plateau. Luo *et al*. proposed, based on CT evaluation, the three‐column concept for the treatment of tibial condyle fractures, where the three columns were medial, lateral, and posterior. The three‐column theory is of great significance to the classification of hyperextension tibial plateau fractures. According to the three‐column classification, fractures can be simple medial column, lateral column, or simultaneous fractures of the medial and lateral columns. Some hyperextension bicondylar fractures involve both the internal and external and posterior columns. At the same time, the posterior slope angle decreases or disappears. Patients in this study were divided into an anteromedial fracture group, an anterolateral fracture group, an anterior internal + anterior external fracture group, and a hyperextension bicondylar fracture group involving three columns based on the three‐column theory.

The results of this study show that in simple anterior tibial fractures, the incidence of tibia anteromedial fractures is highest, followed by anterolateral fractures. The anteromedial tibial fracture is relatively complete. Anterolateral tibial plateau fractures are manifested as anterolateral compression, and they are often combined with splitting fractures of the anterolateral tibial wall. In this group of 7 patients, there were 4 cases of tibial anterolateral compression combined with anterolateral wall splitting and 3 cases of simple anterolateral compression. In this group, 3 cases of fractures of the anterior tibia plateau involved both the anteromedial and anterolateral sides of the tibia, and the affected areas of the 3 cases were all located behind the patellar tendon. Hyperextension three‐column fractures are a relatively common type of hyperextension tibial plateau fracture. This type of hyperextension injury involves the anteromedial and anterolateral columns of the tibial plateau, causing compression fractures of the anterior plateau, and is combined with loss of the posterior slope of the tibial plateau^17^.

Hyperextension tibial plateau fractures are often accompanied by soft tissue (ligament and meniscus) injuries. Ali *et al*.^18^ reported that 52% of 25 patients with hyperextension injuries were in the posterior joint capsule, 40% had ACL injuries, 20% had meniscus injuries, and 16% had medial and lateral collateral ligament and posterolateral horn injuries. Chiba *et al*.^19^ reviewed 12 cases of hyperextension tibial plateau fractures, 11 of which had severe ligament injuries. In Gonzalez's 15 cases of hyperextension injury, 27% of patients had soft tissue‐related injuries, including ligament and meniscus injuries, while only 4% of patients in the injury group had soft tissue‐related injuries with other complex mechanisms^20^. The present study determined that simple anterior tibial fractures involve more diagonal injuries, leading to combined ligament injuries. Simple anterior tibial fractures have a higher incidence of combined ligament injury, and are often accompanied by PLC injury, medial collateral ligament injury, or rupture of the ACL and PCL. In these injuries, the knee joint is unstable. In patients with two‐column or three‐column fractures, the fracture absorbs most of the energy, so the possibility of soft tissue damage is relatively small. Such fractures are prone to complications of the soft tissues, such as compartment syndrome, popliteal blood vessels, and tibial nerve injuries, which require clinical attention. There were 2 cases of common peroneal nerve injury and 2 cases of compartment syndrome in this group.

### Choice of Surgical Approaches for Hyperextension Anterior Tibial Plateau Fractures

For hyperextension tibial plateau fractures, the choice of treatment incision for anterior tibial plateau fractures should be slightly anterior, and use of a medial/lateral approach or a medial–lateral approach is determined according to the fracture location^6^. For simple anterior tibial fractures, anteromedial or anterolateral fractures can be exposed satisfactorily by anteromedial or anterolateral approaches, respectively. However, if the compression is located behind the patellar tendon, due to the small mobility of the patellar tendon, it is difficult to fully expose the fracture through a simple anterior internal or external incision^21^. Using the modified anterior median approach, a longitudinal incision is made on the lateral side of the tibial tubercle. The fracture site can be fully exposed through both sides of the patellar tendon, and the fracture can be reduced and fixed under direct vision. However, this approach is more risky and can easy cause skin and soft tissue necrosis and infection. The patients in this group in whom the median approach was used did not have wound infections or skin necrosis. This low infection rate may be related to our strict control of indications, gentle protective flap surgery, and limited exfoliation range^22^. We have also ensured that there was adequate drainage and moderate pressure dressing, which can reduce the occurrence of subcutaneous hematomas. For hyperextension three‐column fractures, due to the serious fractures involving the medial, lateral, and posterior columns of the tibial plateau, surgical treatment is required with both medial and lateral incisions. Attention should be paid to the distance between the skin bridges.

### Fixation strategy for hyperextension anterior tibial fractures

Fractures of the anterior edge of the tibia caused by hyperextension injuries are compression fractures, and the height of the articular surface needs to be restored during the operation. According to Kfuri *et al*.^10^, if the depression involves the edge, the edge should be reconstructed in this specific quadrant to restore the stability of the joint. For severely damaged hyperextension three‐column fractures, not only does the anterior tibial fracture need to be reduced, but the posterior inclination of the tibia also needs to be restored. Anterior medial and anterior lateral fixation are often used. Because there is no special plate for the anterior medial fixation, a The Tomofix plate designed to be used in high tibial osteotomy is used for the anterior medial fixation of the tibia in our group. For simple anteromedial tibial fractures, there is often a relatively complete fragment. The operation is gentle, keeping the fracture intact and avoiding fragmentation. It is usually fixed with a T‐shaped locking plate. Some patients with anterolateral fractures are simply anterolateral compressions, and some patients also have split fractures of the anterolateral tibia. When combined with split fractures of the anterior lateral wall of the tibia, the lateral fracture was opened in a book‐like manner, then the anterior lateral collapsed fracture was reduced under direct vision. Bone grafting maintained the reduction of the fracture, and then the lateral fracture was reduced and fixed with an L‐shaped plate.

There is no dedicated plate fixation system for simple anterolateral marginal compression fractures and posterior tibial collapsed fractures of the patellar tendon. In this group of patients, 3 cases of simple anterolateral tibia collapse and 3 cases of anteromedial + anterolateral (posterior to the patellar tendon) were fixed with horizontally‐oriented rim plates. Because the fracture site is blocked by the anterior patellar tendon, it is difficult to reduce and fix the fracture. Bermúdez^23^ reports on the application of a horizontal strip plate for the treatment of complex tibial plateau fractures. He used a traditional reconstruction plate that was shaped according to the shape of the tibial plateau, placed horizontally on the joint edge of the tibial plateau, and fixed on different planes with multiple screws through the plate. This method has been used to assist in the treatment of peripheral fractures of the distal femur, the ankle joint, and the proximal humerus. In the case of tibial plateau fractures, because the posterolateral fracture is blocked by the fibula, it is difficult to fix the fracture. Cho used a horizontal plate to fix the fracture through the anterolateral approach, avoiding the use of the posterior approach to treat this type of fracture, and obtained a satisfactory clinical effect^24^. He used a plate that was shaped and attached along the joint edge and then fixed. Biomechanical studies have also confirmed that although the absolute biomechanical strength of the horizontal plate fixation is lower than that of the support plate, it can achieve a good fixation strength, which is sufficient for early functional exercise of the affected knee^21^, ^22^, ^23^, ^24^, ^25^. In this group of patients, a 2.7‐mm miniature reconstruction locking plate was used to fix the anterior tibia after shaping. There was no loss of fracture reduction at the anterior edge of the tibia after the operation.

In summary, hyperextension anterior tibial plateau fractures can be divided into simple anterior tibial fractures and hyperextensive three‐column fractures combined with anterior fractures. Simple anterior tibia fractures include anteromedial, anterolateral, and anteromedial + anterolateral tibia fractures. The simple anterior tibial plateau fracture is prone to “diagonal” injury, and the incidence of knee ligament injury is high. The hyperextension three‐column tibial plateau fractures were combined with loss of posterior slope of the tibial plateau. Anatomical reduction of fractures of the anterior edge of the tibia helps to restore the stability of the knee joint; therefore, surgical reduction and internal fixation are required. In this study, the appropriate surgical approach and fixation method were selected based on the different positions of the anterior tibial fracture and satisfactory results were obtained after surgery.

A limitation of this study is that it was a retrospective analysis. There are few cases and there is no control group for other approaches. Long‐term efficacy and complications need to be further studied and analyzed. Comprehensive analysis based on the fracture characteristics and condition of soft tissues is also necessary.
